# Solitary Sign of Third Nerve Palsy in a Conscious Patient With Epidural Hemorrhage

**DOI:** 10.7759/cureus.10003

**Published:** 2020-08-24

**Authors:** Praveen Kumar Gupta, Mohammad Arif, Likhita Shaik, Romil Singh, Kaushal Shah

**Affiliations:** 1 Neurological Surgery, Jawaharlal Nehru Medical College, Ajmer, IND; 2 Internal Medicine, Ashwini Rural Medical College Hospital and Research Centre, Solapur, IND; 3 Medical Oncology, Mayo Clinic, Rochester, USA; 4 Internal Medicine, Metropolitan Hospital, Jaipur, IND; 5 Psychiatry, Griffin Memorial Hospital, Norman, USA

**Keywords:** third nerve palsy, epidural hemorrhage, brain trauma injury

## Abstract

Epidural hematoma is a life-threatening complication of head injury, which often occurs as a result of blunt trauma to the skull. Unregulated hematoma expansion in any setting results in elevated intracranial pressure and may contribute to the compression of the oculomotor nerve among several other adversities culminating in various long-lasting complications in the future. In this case report, we present the findings of a rare, insightful case of a 47-year-old Southeast Asian male with no established prior medical history apart from being a victim of blunt trauma attributable to a fall four days before presenting to the emergency department with abrupt onset of diplopia and drooping of the left eyelid. The initial physical examination helped to establish a diagnosis of third nerve palsy. A non-contrast CT of the head was conducted, and its findings revealed the presence of a right temporal-parietal-occipital epidural hemorrhage, with no mass impact on the cerebral hemisphere. The patient later underwent a successful left temporoparietal craniotomy, during which 100-125 ml of blood was drained out. Post-surgery, a near-full reduction of ptosis was recorded at the end of the first week. This case report summarizes this ingenious depiction of a partial third nerve palsy presenting as the sole sign of the epidural hemorrhage in a cognizant patient.

## Introduction

Intracranial hemorrhages are categorized as epidural or extradural, subdural, subarachnoid, and intraparenchymal, depending on their location in the brain. The epidural and subdural hemorrhages commonly occur as a result of trauma, while the subarachnoid and intraparenchymal bleeds occur due to associated cardiovascular risk factors such as age, hypertension, diabetes, and hyperlipidemia. An epidural hemorrhage arises from a bleed from the middle meningeal artery into the space between the dura mater and the skull. The incidence of epidural hemorrhage occurs in 2% of head injury cases [1]. It usually presents a classic momentary loss of consciousness after the injury followed by a pseudo-recovery period known as the lucid interval and again followed by a deterioration of the clinical status. Subdural hemorrhage comprises 5% to 25% of head injury cases with an injury to the bridging veins in between the dura and arachnoid mater. Any brain hemorrhage can present with a broad spectrum of non-specific symptoms, such as headache, nausea, vomiting, confusion, decreased consciousness, lethargy, motor deficits, aphasia, seizure, or personality changes [1,2].

Subarachnoid hemorrhage commonly occurs due to aneurysm rupture or an arteriovenous malformation into the potential space between the pia and arachnoid mater. Non-aneurysmal subarachnoid bleeds occur after trauma with a blunt head injury with or without penetrating trauma or sudden acceleration changes to the head. This bleeding occurs in about 5% of patients presenting with strokes. Classic severe thunderclap headache and nuchal rigidity are its key features. Intraparenchymal hemorrhage is due to bleeding from an artery or vein that supplies the brain parenchyma. It accounts for 10% to 20% of cases that present with stroke. The prime features of intraparenchymal hemorrhage are stroke-like lethargy, weakness, slurred speech, syncope, vertigo, or changes in sensation account for its presentation in addition to the above common symptoms of intracranial bleeds [3,4].

The incidence of an acquired oculomotor or third nerve palsy (TNP) as reported by a population-based study was 4 per 100,000. Vascular ischemia, trauma, intracranial neoplasms, hemorrhage congenital, and idiopathic conditions consider for the various causes of TNP, with microvascular and trauma accounting for a majority [5,6]. When associated with trauma, other cranial nerve palsies and neurological deficits are common presentations. An isolated TNP without any other neurological signs is a rare condition after closed head trauma [7]. Clinically it presents with an inferior and lateral rotation (down and out) of the affected eyeball and associated ptosis and mydriasis [8]. 

Furthermore, a partial TNP that spares the autonomic fibers and does not involve an internal ophthalmoplegia is found infrequently. Cases can be termed as complete or partial, depending on if all or only specific findings are present. A partial TNP case with an extradural hemorrhage in an awake patient without any other neurological signs or symptoms has never been reported in the literature. It is quite challenging to diagnose such conditions in the absence of other neurological deficits [9]. Here, we present an uncommon and atypical case of head trauma where a partial TNP is the only presenting sign of a traumatic brain injury in an awake patient.

## Case presentation

**Figure 1:**
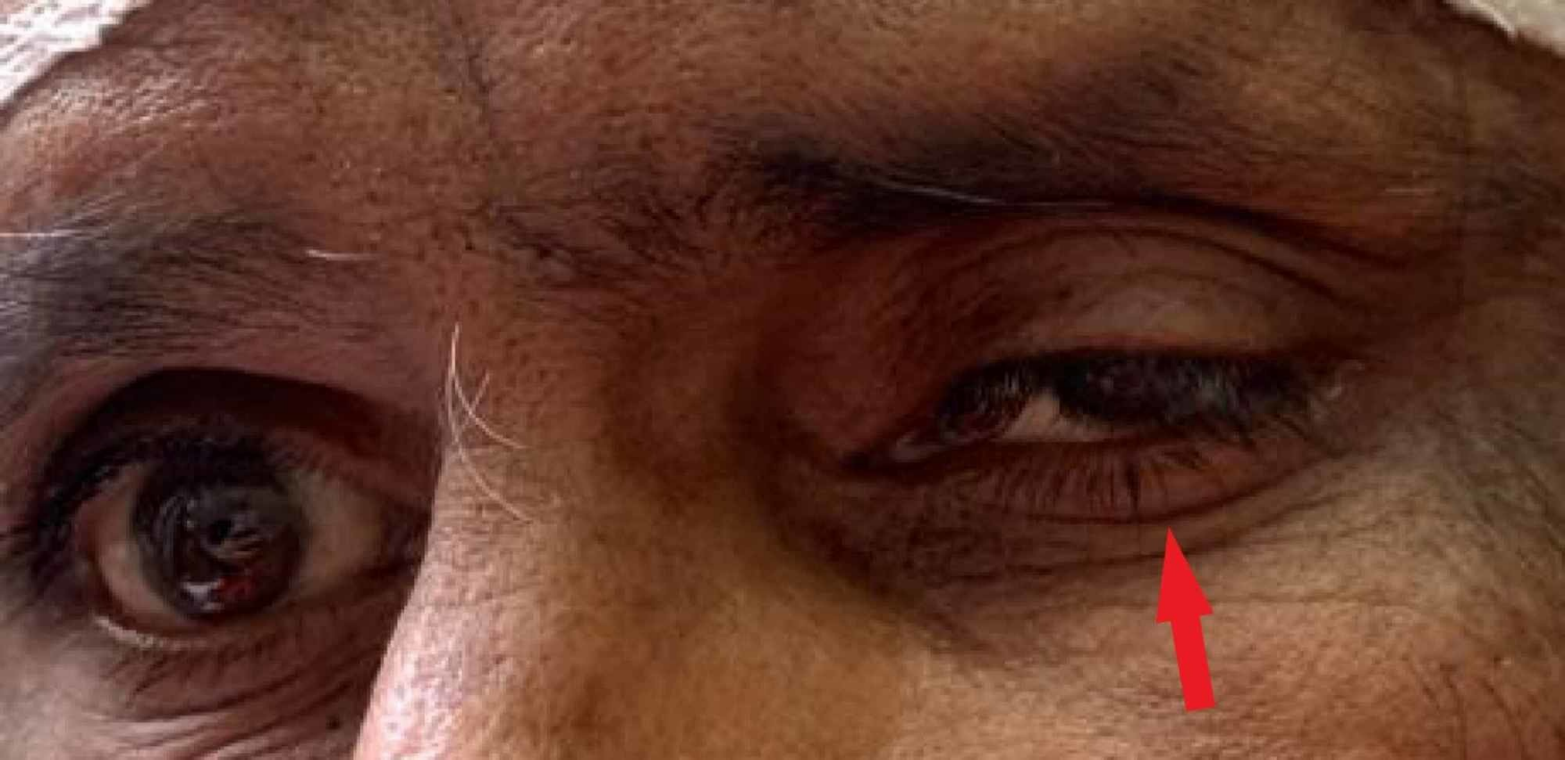
Ptosis of the left eye

A 47-year-old Southeast Asian male presented to the emergency department with sudden onset of the diplopia and drooping of the left eyelid (Figure 1). 

He has no significant past medical history. However, the patient reported a fall with mild blunt head trauma four days ago. The patient was asymptomatic with no neurological deficits during or immediately after the fall. He also did not experience loss of consciousness, seizures, or vomiting. His smoking history was negative. The patient had no diagnosed significant medical or family conditions and was not taking any anticoagulants or antiplatelet agents. 

**Figure 2:**
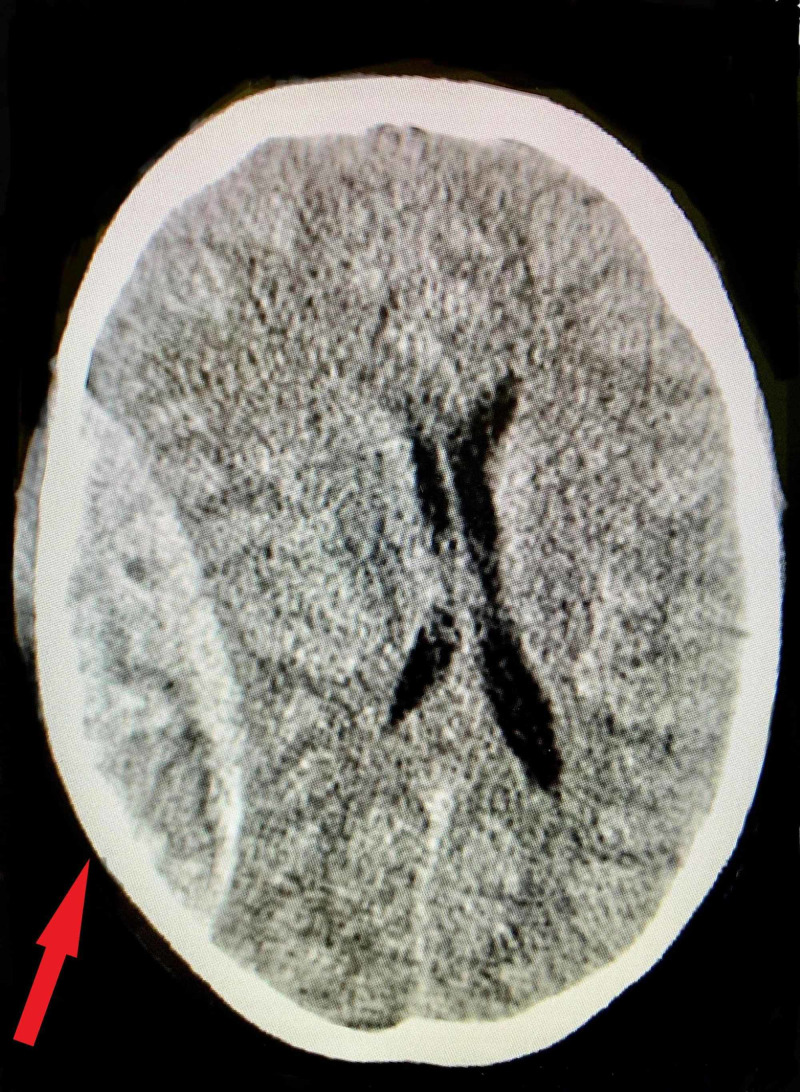
Epidural hemorrhage

On initial assessment, he was fully conscious and well oriented to time, place, and person. Physical exam revealed complete ptosis of the left eyelid. Bilateral light reflexes and extraocular movements were normal. The rest of the neurological and cardiopulmonary examination was normal. A diagnosis of the partial oculomotor nerve palsy was established based on the clinical presentation. A non-contrast CT of the head was subsequently ordered, and indicated that a right temporal-parietal-occipital epidural hemorrhage was noted (Figure 2). No signs of mass effects on the cerebral hemisphere. It was also associated with a small contusion in the frontal region. Other investigations to rule out bleeding disorders and metabolic causes were negative. 

The patient underwent left temporoparietal craniotomy. About 100-125 ml of blood was drained out. No source of bleeding was found. At the end of the first week postoperatively, near-complete improvement in ptosis was achieved.

## Discussion

Extradural or epidural hemorrhage is the collection of blood between the dura mater and the inner table of the skull. Most of them develop as a result of middle meningeal arterial bleed with a minor contribution from the dural venous sinus. Immediate loss of consciousness followed by a pseudo-recovery phase is defined as the lucid interval, and again, a rapid deterioration in the neuroclinical status is a common finding. This course correlates with the rapid expansion of the hematoma and stabilizing over the period of time. It can lead to a rise in the Intracranial tension (ICT) and eventually lead to various cranial nerve compressions, especially the third cranial nerve, also known as oculomotor nerve [10,11]. 

The oculomotor nerve can be damaged anywhere during its course. It originates in the midbrain, intramedullary part, and paths through the posterior petroclinoid ligament, cavernous sinus (intracavernosal), and supraorbital fissure to enter the orbit to the intra-orbital region. Somatic fibers of the TNP that supply the eye muscles and the levator palpebrae superioris that control extraocular movements and elevate the upper eyelid lie in the deeper parts of the nerve, while the autonomic fibers that control the pupillary miosis and curvature of the lens lie on the superficial aspect [7,10].

The nerve transection or a compression evidenced by an adjacent bleed contributes to the majority of complete oculomotor palsies presenting with the classic down and out eyeball, mydriasis, and ptosis. An increase in the ICT can also cause TNP via transtentorial herniation. However, increased ICT has been ruled out in our patient by the presence of normal fundoscopy findings. Injury at the petroclinoid ligament, followed by a diffuse axonal injury, is a hypothesized cause of rare forms of isolated complete TNP [10,12,13]. 

Neuroimaging plays a critical role in the diagnosis of these life-threatening conditions that present with minimal neurological signs [14]. Though standard imaging studies such as CT and MRI cannot always correlate with the clinical status, clinicians must proceed to perform other advanced investigations like angiography or orbital CT in order to have a better understanding of the condition. Likely, a precise mechanism of damage cannot be attributed to the observed deficits because diagnostic tests are not completely sensitive to capture the immediate post-traumatic changes [15].

In our patient’s case, isolated post-traumatic oculomotor nerve palsy without internal ophthalmoplegia was clinically diagnosed on the left side after closed head trauma. The hematoma that occupies over a large area of temporal, parietal, and occipital regions has a role in straining or compressing the cranial nerve III along the edge of the tentorium, leading to intact mental status and symptoms of ipsilateral, partial TNP. However, it remains a pathophysiologic conundrum as to why only the somatic fibers that lie on the deeper aspect were affected. Inadequate literature on the mechanisms that cause partial TNP in an otherwise clinically stable and conscious head injury patient warrants further probing and research. Furthermore, patients presenting with isolated TNP must undergo brain imaging to rule out any forms of a brain hemorrhage [14,15]. Physicians must be notified of this rare but treatable presentation of isolated TNPs, as early intervention results in complete recovery as occurred in our case. 

## Conclusions

Patients presenting with TNP should be investigated thoroughly to identify precise etiology. It is crucial to rule out epidural hematoma even when the patient presents with atypical signs, including the sole symptom of TNP. In an unusual presentation with no initial suspicion of intracranial hemorrhage, all patients should be adequately evaluated based on the recent trauma history and imaging studies. Provided the life-threatening consequences of untreated epidural hematoma, all healthcare providers must assess TNP meticulously.
